# *UnityMol* prototype for FAIR sharing of molecular-visualization experiences: from pictures in the cloud to collaborative virtual reality exploration in immersive 3D environments

**DOI:** 10.1107/S2059798321002941

**Published:** 2021-05-28

**Authors:** Xavier Martinez, Marc Baaden

**Affiliations:** a CNRS, Université de Paris, UPR 9080, Laboratoire de Biochimie Théorique, 13 Rue Pierre et Marie Curie, 75005 Paris, France; b Institut de Biologie Physico-Chimique–Fondation Edmond de Rothschild, PSL Research University, Paris, France

**Keywords:** molecular visualization, FAIR sharing, COVID-19, virtual reality, collaborative environments

## Abstract

Motivated by the current COVID-19 pandemic, which has spurred a substantial flow of structural data, the use of molecular-visualization experiences to make these data sets accessible to a broad audience is described. Using a variety of technology vectors related to the cloud, 3D and virtual reality gear, how to share curated visualizations of structural biology, modeling and/or bioinformatics data sets for interactive and collaborative exploration is examined. FAIR is discussed as an overarching principle for sharing such visualizations. Four initial example scenes related to recent COVID-19 structural data are provided, together with a ready-to-use (and share) implementation in the *UnityMol* software.

## Abstract 2

Visualization renders structural molecular data accessible to a broad audience. An approach to share molecular-visualization experiences based on FAIR principles is described. The workflow is exemplified with recent COVID-19-related data.

## 1. Introduction   

The COVID-19 pandemic has spurred a wealth of new structural biology data related to the severe acute respiratory syndrome coronavirus SARS-CoV-2 and its interactions with other macromolecules involved in the development and spread of the disease (Baker, 2020 ▸; Dömling & Gao, 2020 ▸). Such structural data are transparent to experts in the field, but require adequate visualization to become accessible to a broader audience. As put by Card *et al.*, ‘visual artifacts aid thought; in fact, they are completely entwined with cognition action’ (Card *et al.*, 1999 ▸). Visualizations prepared by experts may serve both educational and research purposes by highlighting key aspects of a given data set or comparing features among several thereby supporting thought and reasoning. Here we aim to experiment in establishing a general framework to prepare such curated educational and research material. We illustrate our point with recent COVID-19 structural and modelling data examples. Our overall goal is to enable easy sharing of a given visual experience with others so that the same content can be accessed in various ways. A particular aim is to treat the sharing of visual experiences with the so-called FAIR principles to render them Findable, Accessible, Interoperable and Reusable (Wilkinson *et al.*, 2016 ▸). These principles should be applied for the underlying raw chemical (Blanke *et al.*, 2020 ▸), structural and modelling data, and it has been pointed out that this is generally the case in crystallography (Helliwell, 2019 ▸). This target is challenging by itself, as common FAIR-based sharing platforms do not provide a category that is well adapted to visual experiences.

Sharing of visualization experiences appears to be a particularly timely topic. The case of the COVID-19 pandemic provides a stringent example for the need to render the latest research data explorable by the broad scientific community. If such data were only to be provided in its raw form, the accessibility would be quite limited. The Worldwide Protein Data Bank (PDB) (Berman *et al.*, 2003 ▸) indeed provides quite a few visualizations along with the actual data files. Recently, the PDB in Europe (PDBe) also proposed a visual analytics platform that is FAIR compliant (PDBe-KB consortium *et al.*, 2020 ▸). Here we try to go one step further in the democratiz­ation of such data by preparing curated visual experiences as a valuable support for cross-disciplinary scientific exchange. In related fields, similar initiatives have recently emerged. Now more than ever, sharing data from molecular simulations and modeling is becoming a priority to ensure reproducibility and accelerate discovery (Amaro & Mulholland, 2020 ▸). An increasing number of initiatives aim to develop open access and reproducible molecular simulations [see for example those by Abraham *et al.* (2019 ▸); Elofsson *et al.* (2019 ▸); Abriata *et al.* (2020 ▸) among other approaches]. Sharing such molecular dynamics (MD) simulation data equally needs efficient visualization and collaboration tools (Tiemann *et al.*, 2017 ▸) for apprehending such data remotely to avoid unnecessary data transfers. By streaming trajectory data upon demand, a systematic transfer of such files, which can easily range between several megabytes to hundreds of gigabytes or more, is avoided. Yet to the best of our knowledge, current initiatives focus on the actual data, not so much on the visualizations and annotation thereof.

Here we present an initial prototype using the *UnityMol* software. We place the focus on collaborative virtual reality (VR) as well as sharing 3D models and scenes to intuitively convey the spatial complexity of these molecular objects. Shape-related features have particular relevance for drug design and hence for expert users, yet they can also be understood by an inexperienced person, in particular through a visual experience such as those described here.

**Figure 1:**
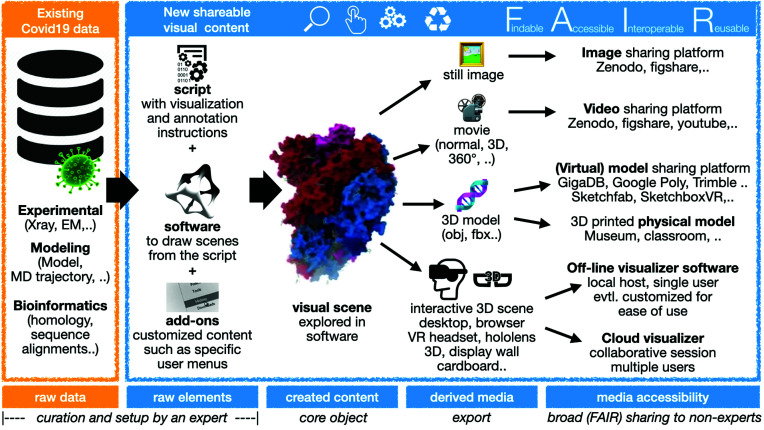
A scheme summarizing the suggested workflow for FAIR sharing of molecular visualization experiences, building on COVID-19-related structural data. Using existing data, new shareable visual content is created from raw elements such as a script with instructions, software to execute the script and eventual add-ons to facilitate the end-user tasks (for instance custom menus to provide only the strictly necessary shortcuts for a given experience). The visual scene that is created can be explored within the software itself or it can be exported in various ways to generate derived media such as images, movies, 3D models and even full experiences in *e.g.* VR contexts. These media can be rendered accessible through platforms that implement FAIR principles and ease their exploration.

The features of the workflow we are seeking to implement are depicted in Fig. 1 ▸. We start from the original raw data that should encompass experimental structural data sets, molecular modelling results and bioinformatics findings. Crystallographic and EM data can be obtained from many portals, such as https://www.covid19dataportal.org/proteins?db=uniprot-covid19. We aspire to prepare new shareable visual content to be set up and curated by an experienced scientist knowledgeable in structural biology. Once prepared, viewing this content could be accessible to a much broader community. To do so, we propose that one could use a molecular visualiz­ation software package to describe the visualization scene and all its annotations through command scripts. The experience can be customized through add-ons, for example providing data-set-specific user menus leading to a visual scene that can be explored directly in the software. This exploration does however require learning at least some basic usage of that software and how to manipulate the scene. This task can be simplified to some extent by designing appropriate simple-to-use custom user menus. As an alternative, the core content to be visualized can be exported to a variety of media such as still images, movies of various formats (possibly stereoscopic ones), native 3D models or a fully interactive scene to be explored with advanced technology. A general framework should support a broad variety of sophisticated hardware such as VR headsets, wall-size stereoscopic displays and the HoloLens, and also simple setups such as Google cardboard or even just running in a web browser. The most expensive among these setups may cost up to several hundred thousand Euro (display wall), headsets are much cheaper (roughly in the 2500–5000 Euro price range), whereas the cheapest options (Google cardboard or equivalent) cost less than 10 Euro, but require a smartphone. To render these media findable, accessible, interoperable and reusable, a variety of options need to be discussed and agreed upon community-wide.

The set of (open source) software plus openly accessible scripts that we propose as a basis to provide such a framework make the visual experience as reproducible as possible (Cohen-Boulakia *et al.*, 2017 ▸). Inherent limitations arise from technical hardware and software dependencies related to rapidly evolving visualization technology that are by nature short-lived with a high rate of change. One way to apply FAIR principles to visualization-centric data is by addressing the setup of the visualization experiences, *i.e.* the underlying software and the related scripts (designated as ‘raw elements’ in Fig. 1 ▸), rather than the resulting media products. A more classical approach is to provide the derived media, which may consist in videos or images for which sharing platforms exist, although this is not enough to optimize FAIR compliance (Weber & Kranzlmuller, 2018 ▸).

We chose the *UnityMol* package to prototype a workflow following the scheme in Fig. 1 ▸ and implemented a proof-of-concept set of visualization experiences. We have been developing and redesigning the *UnityMol* software, which provides specific advantages for the tasks at hand in its latest version (Lv *et al.*, 2013 ▸; Laureanti *et al.*, 2020 ▸). Many alternative software options of high value could be considered, such as those recently reviewed for protein visualization (Martinez *et al.*, 2020 ▸). Particularly relevant packages in the opinion of the authors include *VMD* (Humphrey *et al.*, 1996 ▸), *PyMOL* (version 1.8; Schrödinger), *Chimera*X (Goddard *et al.*, 2018 ▸), *JSmol* (Hanson, 2010 ▸) and Mol* (Sehnal *et al.*, 2018 ▸). We opted for *UnityMol*, our own package, for proof of principle and to make use of supported media and technology vectors combined with the relatively simple and single-window UI design with only a few menus to operate.

Our ambition is to provide a few proof-of-concept implementations that others can subsequently build on to further improve or simply adapt them. We have implemented all of the functionalities that we deemed necessary to prepare the COVID-19-related materials, and present here four basic examples. Based on these example scenes, we experiment with applying FAIR principles to such visualization-centric data.

Concerning the specific case of COVID-19, many data portals and hubs have been put together to provide more unified access to the relevant data. A brief selection are listed in Table 1 ▸ and served as a testing ground for our visualization experiments. Many of the listed portals provide very direct access to the data that we require for our visualization experiences, while a few provide more general search engines with a less direct path to the actual data set of interest. We did not include the classical routes to search data such as the PDB, GigaDB (Sneddon *et al.*, 2012 ▸; Xiao *et al.*, 2019 ▸) or Zenodo, and we only included one of the many relevant initiatives by individual scientists or laboratories as an example.

## 2. Materials and methods   

Here, we will briefly describe the key functionalities implemented for the purpose of sharing molecular-visualization experiences. More detailed technical information is provided on github through the project overview page (https://github.com/bam93/fair_covid_molvisexp/tree/master/overview). As an overarching principle, we want to be able to generate a broad variety of media, including still images and videos, based on these visual scenes describing the core of the contents, capturing them from within the running software, ideally in an executable build and, for a handful of technically more involved features, from within the *Unity*3*D* editor environment. For this purpose we implemented, extended and stabilized several features. A certain number of those features are still only experimental.

We added a Python console and API for scripting within *UnityMol*, providing an easy way to formalize and share the narrative of a given experience, as well as facilitating the reproducibility of all necessary steps leading to a scene. The interactive Python console can be toggled on and off, appearing on the right-hand side of the screen. Scripts can be loaded from the console or through a UI menu button. We furthermore implemented the possibility of generating custom user menus so that complex and fine-tuned functionality can be achieved with a one-click action. The Python-based menu uses the zmq and tkinter modules and needs to be started separately from the command line. A possible practical use could be to group shortcuts to relevant structural ensembles in the light of COVID-19 for visual analysis, comparison or even manipulation. The latter can be achieved through interactive simulations such as interactive molecular dynamics (IMD). In such simulations, the user may actively manipulate the molecular system and steer the exploration of conformational space to interesting regions by adding custom forces to the simulation. *UnityMol* implements the generic IMD protocol for steering molecular simulations through *MDDriver* (Delalande *et al.*, 2009 ▸), but also provides a few in-app implementations such as a rigid protein–protein docking module.

The scenes can be annotated by the user through several functionalities such as text bullets or free-hand drawing. A guided-tour function has been implemented to walk the user through a set of predetermined key frames in a fully interactive experience. These features are demonstrated in two supplementary movies, which are accessible through the Zenodo and figshare pages.

**Figure 2:**
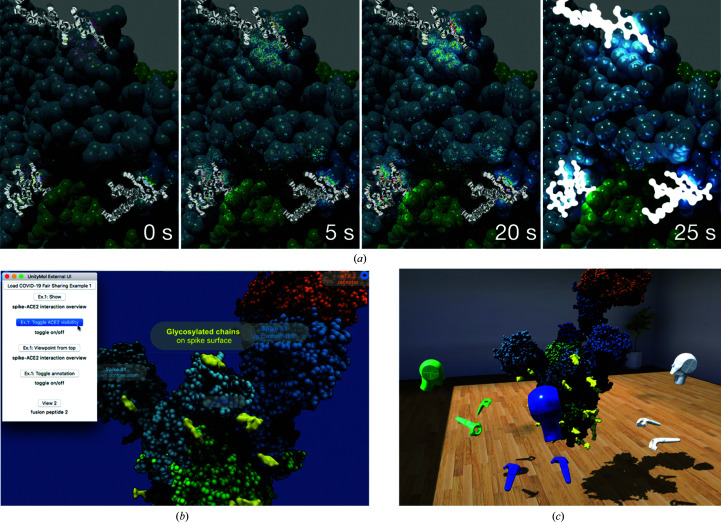
Ilustration of three types of visual experiences within *UnityMol*. (*a*) Interactive raytracing of a simple structural view of the spike–ACE2 complex model derived from cryo-EM experiments (example 1). A series of four images at distinct time intervals is shown to depict the ongoing work of the raytracer. After 25 s a denoiser pass is executed, significantly increasing the visual clarity of the scene. The raytracer indefinitely continues to improve the scene. The fluidity and interactivity are only limited by the CPU-bound performance. The indicated timing was obtained with a 2013 3.5 GHz 6-Core Intel Xeon E5. (*b*) A custom menu extension for example 1, providing the possibility of toggling annotations on and off along with direct access to different representations and viewpoints. (*c*) Screenshot of a collaborative multi-user session where three participants are examining example 1 with VR headsets and controllers.

A particular focus concerns the possibility of generating effective high-quality graphics, for instance in order to convey the complex shapes of molecular surfaces using ambient occlusion (AO) or to draw the user’s attention to a certain part of the structure of interest using photographic effects such as depth of field (DOF). In addition to the molecular structures themselves, their properties (such as electrostatic field) can also be depicted (Laureanti *et al.*, 2020 ▸). In the latest version of the software we added an experimental feature enabling interactive CPU-based raytracing using the *OSPRay* package (Wald *et al.*, 2017 ▸); GPU raytracing is not yet fully implemented. This is illustrated for our first example of the spike trimer complex with angiotensin-converting enzyme 2 (ACE2) from cryo-EM data (Fig. 2 ▸ *a*). We added a custom menu to simplify navigating through this example (Fig. 2 ▸ *b*). We can also explore the scene collaboratively in a multi-user VR session (Fig. 2 ▸ *c*), where avatars represent each participant. Each participant has an individual point of view, separate from each other, and sees the avatars of all other users along with the molecular objects of the scene. Participants may choose to group together to adopt a similar viewing angle. To capture such sessions, still images from screenshots or movies from video capture can be created at user-defined resolution. In general, however, image and video exports do not preserve the full complexity of the initial object. For this purpose we added the export of textured 3D polygonal objects in the common obj and fbx formats and will probably soon add glTF format. Such 3D models can be transformed into tangible physical objects through 3D printing (Gillet *et al.*, 2005 ▸). These media can be generated and exported with specific options such as a 360° view or a 3D stereoscopic enhancement. For these purposes, specific cameras had to be added within *Unity*. Special effects such as AO, DOF and depth cueing can currently only be exported partially.

## 3. Results   

We have started the process of building up a collection of COVID-19 example scripts with several objectives: they should be easy to understand, even for nonspecialists, others should be able to re-run them under various conditions in terms of visualization hardware and modality, and (in the future) it should be simple to use them as a starting scene for shared multi-user sessions. A particular emphasis is placed on the design of visual experiences suitable and tested for VR exploration, as our current research is focused on this technology. In our opinion its potential is still largely underexplored in academia and industry, be it for research, collaboration or education purposes (Morrison, 2016 ▸). The results section is based around the stages of the pipeline depicted in Fig. 1 ▸.

### 3.1. Raw data and targeted visual experiences   

To illustrate the sharing of such visual experiences, we have prepared four typical data sets, depicted in Figs. 2 ▸ and 3 ▸: a simple structural view, a collection of small molecules binding to a viral protein, an MD trajectory and a bioinformatics sample with conservation as well as mutation data.

The first example, depicted in Fig. 2 ▸, aims at setting up a simple structural view of the SARS spike glycoprotein complex with human ACE2. The model we use is derived from cryo-EM density measurements and has been deposited in the PDB. This trimeric complex, accessible as PDB entry 6cs2 (Kirchdoerfer *et al.*, 2018 ▸), was used as the starting point for many early SARS-CoV-2 studies.

**Figure 3:**
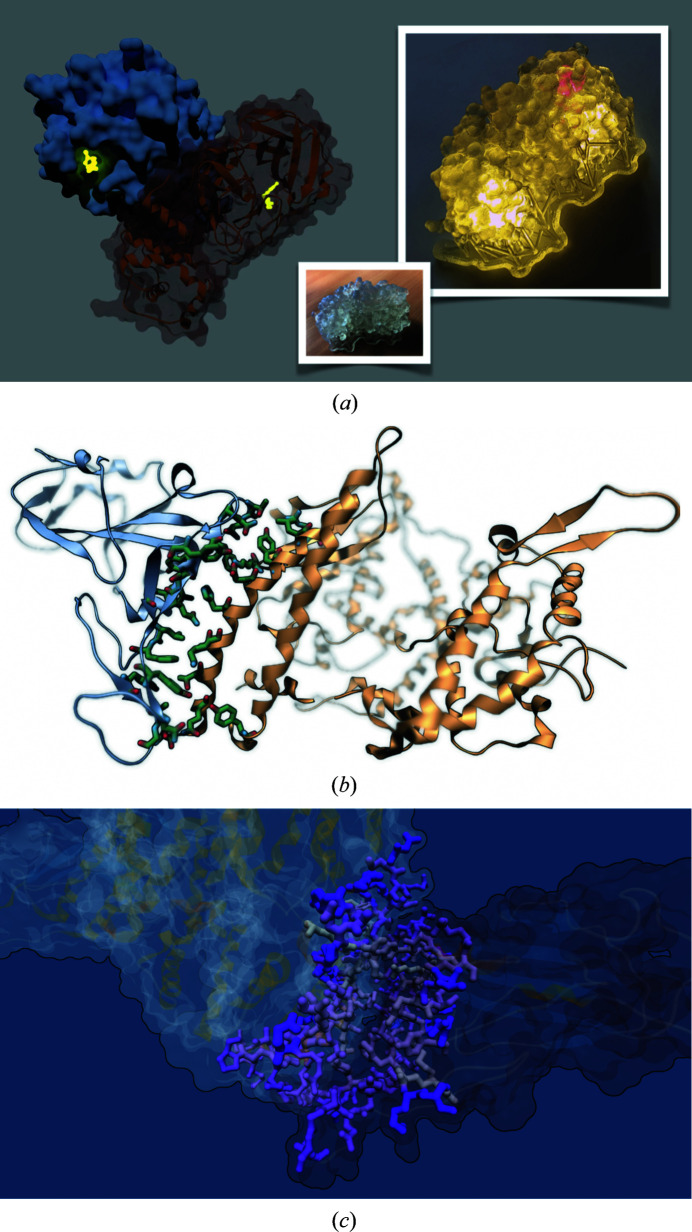
A glimpse into examples 2–4. (*a*) Example 2 depicts drug molecules (shown in glowing yellow) binding to the dimeric biological assembly of the COVID-19 main protease (monomers shown as a blue surface and brown cartoons) in 92 PDB structures determined by X-ray crystallo­graphy. The image is a snapshot taken from an animation of the whole data set. The insets show a 3D-printed translucent monomer model (bottom) and how one may illuminate it (right) to highlight specific areas with a laser (red spot). The orientation of the image on the right is similar to that of the brown monomer subunit shown on the left. (*b*) Example 3 renders a molecular-dynamics simulation of the SARS-CoV-2 spike receptor-binding domain binding to the ACE2 receptor (D. E. Shaw Research). A depth-of-field effect is used to draw attention to the binding interface. (*c*) Example 4 represents results on species variability of the binding interface from a bioinformatics analysis. The interacting residues are colored on a pink color scale according to the *HADDOCK* docking score from Rodrigues *et al.* (2020 ▸) and their size is varied according to residue conservation as assessed through a Shannon entropy measure.

We then have a comparative look at where drug molecules bind to the main protease of COVID-19 in our second example (Fig. 3*a* ▸). The visualization is inspired by an animation of small molecules in 92 PDB structures (https://www.rbvi.ucsf.edu/chimerax/data/sars-protease-may2020/) prepared by the *ChimeraX* team (Goddard *et al.*, 2018 ▸). All of the PDB structures were obtained by X-ray crystallography (the PDB codes can be found in the scripts that we provide). We also created a 3D print of the protease monomer.

Molecular-dynamics simulations provide another inestimable resource for insight into molecular mechanisms. Example 3 (Fig. 3*b* ▸) is based on a trajectory depicting a binding event of the receptor-binding domain (RBD) of the SARS-CoV-2 spike protein and the human ACE2 receptor (DESRES-ANTON-10857295 and DESRES-ANTON-10895671; D. E. Shaw Research; https://www.deshawresearch.com/downloads/download_trajectory_sarscov2.cgi/). The underlying structural data were determined by X-ray crystallography (PDB entry 6vw1).

The fourth example illustrates a visual experience related to representing the results of bioinformatics analyses. Our example is built upon the freely available data from a recent study on cross-species transmission of SARS-CoV-2, highlighting the species variability of viral–host protein inter­actions (Rodrigues *et al.*, 2020 ▸). We visually map this data onto the cryo-EM-derived ACE2–RBD complex structure (Fig. 3*c* ▸).

### 3.2. From raw elements to created content   

**Figure 4:**
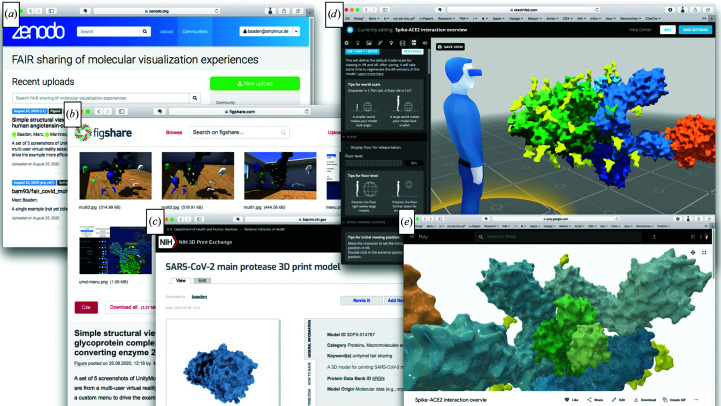
A visual overview of content-sharing platforms hosting our visual experiences to make them FAIR-compliant. (*a*) depicts a Zenodo community regrouping all of the content created for this work (https://zenodo.org/communities/fair-molvisexp/). (*b*) is a figshare collection of a subset of this content (https://doi.org/10.6084/m9.figshare.c.5101400). (*c*) illustrates the entry for our 3D-printed model from Fig. 3 ▸(*a*) in the NIH 3D Print Exchange (https://3dprint.nih.gov/discover/3DPX-014787). (*d*) illustrates a 3D model uploaded to Sketchfab (https://skfb.ly/6UFOw) with the possibility of editing AR and VR viewing settings, as the platform automatically enables access to mobile-phone and desktop VR rendering in addition to web-based exploration. (*e*) shows the same 3D model rendered in the Google Poly web-based explorer (https://poly.google.com/view/5zsJiglTWbm). (*c*), (*d*) and (*e*) are accessible through the respective referenced 3D model data sets on Zenodo, an stl file for 3D printing and an fbx file for 3D model building.

The first level of sharing these four examples is to make the software and the scripts themselves available. We considered the Zenodo and GigaDB platforms, and chose Zenodo as the main platform for our experiment. Typically, no dedicated category for ‘visual experiences’ exists in these databases, whereas the classical categories such as software, data set, image, video, workflow or other only partially reflect this case. As a first approach, we created a Zenodo community (Fig. 4*a* ▸) for the FAIR sharing of molecular-visualization experiences at https://zenodo.org/communities/fair-molvisexp/. We then linked a github repository for our visualization scripts to Zenodo and the collection. Upon execution of the raw elements, the scripts that we designed implement the targeted visual experiences for the four example systems described above, thereby giving access to the created content. This step cannot formally be decoupled from the derived media described in the next paragraph, as the chosen medium defines how the scientist intends to ‘consume’ the created contents. Some scripts may allow the generation of a variety of media from a single source, while other scripts may be specific to a certain medium. This aspect is dictated by technical constraints.

### 3.3. Derived media and media accessibility   

We produced a set of derived materials, including pictures, movies and 3D objects. Cloud platforms for pictures and movies are very common nowadays and we will not go into much detail on the media accessibility for these derivatives. We used both the Zenodo and figshare platforms (http://figshare.com) for pictures and for movies, the former for consistency and the latter because of its visually oriented interface and popularity. To regroup our productions on figshare, we created the collection (https://doi.org/10.6084/m9.figshare.c.5101400) shown in Fig. 4(*b* ▸). We also experimented with tangible physical objects as a particularly accessible form for 3D models through 3D printing. Many generic 3D-printing databases exist, whereas the NIH 3D Print Exchange (Coakley *et al.*, 2014 ▸) is a specific research initiative dedicated to bioscientific 3D prints. We deposited our models in this resource (Fig. 4*c* ▸). Such 3D models can also be disseminated and shared virtually, which is a less common process. We experimented with four platforms: Sketchfab (https://sketchfab.com; Fig. 4*d* ▸), Google Poly (https://poly.google.com; Fig. 4*e* ▸), SketchBoxVR (http://sketchboxvr.com) and Trimble 3D Warehouse (https://3dwarehouse.sketchup.com). For the time being, we used the data-set, software, figure and video categories to reference all information on Zenodo. We have created a versioned catalog page of all of the outcomes of these experiments at https://github.com/bam93/fair_covid_molvisexp/tree/master/overview. Once all of the functionalities described here have been stabilized in the *UnityMol* software, we will provide instructions on how to export to these platforms on the same catalog page.

### 3.4. Challenges   

Referencing our data sets on these platforms only represents the first, relatively easy step towards implementing the FAIR principles. One inherent challenge is that some of the elements we share require a specific program to view, for instance the command scripts that run with *UnityMol*. Such a dependence is difficult to reconcile with the FAIR principle, in particular in terms of interoperability and reusability. For interoperability, the developers of other visualization software would need to implement a compatibility layer to interpret such command scripts and maintain them, which may not be a viable and easily adoptable generic solution. Reusability is hampered by the fact that software solutions are relatively short-lived and typically become obsolete within a few decades. Some technical workarounds such as virtual machines may exist, but may themselves be subject to such obsolescence. Further issues may lie in the interoperability and reusability aspects for the (meta)data associated with the visual experiences. For example, metadata are needed to describe software version, script purpose, underlying data sets, dependencies and the variants of output that can be produced. There are definitely many ways to go about implementing the FAIR principles. In this early attempt at visual experiences, we first and foremost want to raise awareness about this as-yet overlooked category, but do not have the ambition to provide a full-fledged solution. Concerning the fact that our data should be findable, the choice to target COVID-19 makes this goal relatively easy to achieve as there are an abundance of hubs for increasing the findability of such data, as described briefly above (Table 1 ▸). The accessible attribute should be taken care of by our choice of well established FAIR-compliant databases for most of the produced media. Interoperability is clearly an area for further improvement. In particular, we consider bridges between different visualization environments as an important way forward. Initiatives fostering universal protocols that can be interchanged, such as MolQL for the query language to define selections (Rose *et al.*, 2018 ▸), need to be extended to describe all ingredients needed for a visual experience, in particular the molecular scene representation. As a modest step in this direction, we have experimented with a *PyMOL* session interpreter for *UnityMol* (https://github.com/LBT-CNRS/PymolToUnityMol), so that *PyMOL* users can reuse their previously prepared scenes. This approach could be taken further by means of a community-agreed and community-supported implementation-agnostic format that would allow the specification of model and map visualizations. Possibly, existing formats such as, for example, the mmCIF format could be extended to include the specification of informative scenes directly within the model file. Reproducibility is intrinsically a difficult aim for visual experiences. However, by providing scripts with full instructions for the setup of the experience and by making the corresponding visualization software available as a versioned open-source project along with ready-to use executable builds for many operating systems and platforms, reproducibility and accessibility are maximized. Furthermore, the software is conceived within a game engine, which by design is easily extensible, with a shallow learning curve.

## 4. Outlook   

From this early experiment, we plan to expand the range of example visual experiences, be it based on the context of the rapidly growing COVID-19 data or more generally the overwhelming structural, modeling and dynamic data of macromolecular assemblies. We are trying to optimize the generated objects so that they can run on devices with limited hardware specifications. An example would be the HoloLens, with good ergonomy but limited graphical power (Müller *et al.*, 2018 ▸), for which we want to be able to export simplified FBX/gltf polygonal models to run smoothly. Most of our efforts will be dedicated to refining the multi-user collaborative features to enable visual sessions for joint exploration.

More generally, our contribution calls for considering whether visual experiences should be assigned a specific category to be added to FAIR sharing platforms in order to take into account the specificities of such interactive, computer-supported graphical representations of research data.

## Supplementary Material

Five simple structural views (still images) of the SARS spike glycoprotein complex with human angiotensin-converting enzyme 2 (ACE2).: https://doi.org/10.5281/zenodo.3999339

Animation of drug molecules binding to SARS-CoV-2 main protease.: https://doi.org/10.6084/m9.figshare.12860069

A 3D model of the spike-ACE2 interaction exported from UnityMol. The raw fbx file can be downloaded, or the 3D model can be directly viewed on Google Poly or on Sketchfab. This also allows viewing in AR/VR with various devices.: https://doi.org/10.6084/m9.figshare.12866981.v2

A 3D model and related files for 3D printing of the SARS-CoV-2 main protease monomer.: https://doi.org/10.6084/m9.figshare.12867314

UnityMol scripts and required files to reproduce the examples described in the paper.: https://zenodo.org/badge/latestdoi/289968174

UnityMol 1.1.1 executable build for MacOSX to run the above scripts.: https://doi.org/10.6084/m9.figshare.12866804

Screen captures illustrating molecular 3D model sharing through Sketchfab, Google Poly and NIH Print Exchange.: https://doi.org/10.6084/m9.figshare.12881606

Screen captures illustrating example 4 (bioinformatics data mapping on a 3D structure).: https://doi.org/10.6084/m9.figshare.12894077

UnityMol 360 degree video with a camera path through the COVID19 spike protein-ACE2 complex.: https://doi.org/10.6084/m9.figshare.12894038

## Figures and Tables

**Table 1:** A few examples of COVID-19 data portals Some of the data in these portals are likely to be cross-referenced or duplicated. The selection of portals is by no means exhaustive; the number of available resources is rapidly increasing.

Name	URL	Relevant data	Comments
EU COVID-19 Data Portal	https://www.covid19dataportal.org	Structures, models	Additional information on sequences, expression data, compounds and targets
COVID-19 Molecular Structure and Therapeutics Hub	https://covid.molssi.org	Structures, models, simulations	Additional information on targets and therapeutics
BioExcel COVID-19 Research	https://bioexcel.eu/covid-19-research/	Structures, models, simulations	Additional information on targets and therapeutics
BioExcel-CV19	https://bioexcel-cv19.bsc.es/#/	Simulations	Web access to atomistic MD trajectories
PDBe-KB COVID-19 Data Portal	https://www.ebi.ac.uk/pdbe/covid-19	Ligand-binding sites, protein–protein interaction residues	A knowledge-base portal with a useful data summary on structures, ligands, interactions, functional annotations and similar proteins
Coronavirus Structural Task Force	https://github.com/thorn-lab/coronavirus_structural_task_force	Structures	A github repository that makes it easy to download and keep an updated copy of the structural data provided
SwissModel	https://swissmodel.expasy.org/repository/species/2697049	Models, interactions	The full SARS-CoV-2 proteome was modeled; dedicated information on interactions is provided
CASP_Commons	https://predictioncenter.org/caspcommons/index.cgi	Models	Generation and evaluation of models of the most challenging proteins and domains of the virus
COVID Moonshot	https://covid.postera.ai/covid/structures	Structures (fragment-oriented)	Links back to other databases such as Fragalysis or RCSB
OpenAIRE COVID-19 Gateway	https://beta.covid-19.openaire.eu	Various research data sets	A search engine referencing many resources
D. E. Shaw Research Resources	https://www.deshawresearch.com/downloads/download_trajectory_sarscov2.cgi	Simulations	
